# Plant Holobiont Theory: The Phytomicrobiome Plays a Central Role in Evolution and Success

**DOI:** 10.3390/microorganisms9040675

**Published:** 2021-03-24

**Authors:** Dongmei Lyu, Jonathan Zajonc, Antoine Pagé, Cailun A. S. Tanney, Ateeq Shah, Nadia Monjezi, Levini A. Msimbira, Mohammed Antar, Mahtab Nazari, Rachel Backer, Donald L. Smith

**Affiliations:** 1 Department of Plant Science, Macdonald Campus, McGill University, Montréal, QC H9X 3V9, Canada; Dongmei.Lyu@mail.McGill.Ca (D.L.); Jonathan.Zajonc@mail.McGill.Ca (J.Z.); Antoine.Page@cnrc-nrc.gc.ca (A.P.); Cailun.Tanney@mail.McGill.Ca (C.A.S.T.); Ateeq.Shah@mail.McGill.Ca (A.S.); Nadia.Monjezi@mail.McGill.Ca (N.M.); Levini.Msimbira@mail.McGill.Ca (L.A.M.); Mohammed.Antar@mail.McGill.Ca (M.A.); Mahtab.Nazari2@mail.McGill.Ca (M.N.); Rachel.Backer@mail.McGill.Ca (R.B.); 2 National Research Council Canada, Aquatic and Crop Resource Development (ACRD), Montréal, QC H4P 2R2, Canada

**Keywords:** phytomicrobiome, plant holobiont, evolution, endosymbiosis, growth promotion, signaling, biocontrol

## Abstract

Under natural conditions, plants are always associated with a well-orchestrated community of microbes—the phytomicrobiome. The nature and degree of microbial effect on the plant host can be positive, neutral, or negative, and depends largely on the environment. The phytomicrobiome is integral for plant growth and function; microbes play a key role in plant nutrient acquisition, biotic and abiotic stress management, physiology regulation through microbe-to-plant signals, and growth regulation via the production of phytohormones. Relationships between the plant and phytomicrobiome members vary in intimacy, ranging from casual associations between roots and the rhizosphere microbial community, to endophytes that live between plant cells, to the endosymbiosis of microbes by the plant cell resulting in mitochondria and chloroplasts. If we consider these key organelles to also be members of the phytomicrobiome, how do we distinguish between the two? If we accept the mitochondria and chloroplasts as both members of the phytomicrobiome and the plant (entrained microbes), the influence of microbes on the evolution of plants becomes so profound that without microbes, the concept of the “plant” is not viable. This paper argues that the holobiont concept should take greater precedence in the plant sciences when referring to a host and its associated microbial community. The inclusivity of this concept accounts for the ambiguous nature of the entrained microbes and the wide range of functions played by the phytomicrobiome in plant holobiont homeostasis.

## 1. Introduction

**Figure 1:**
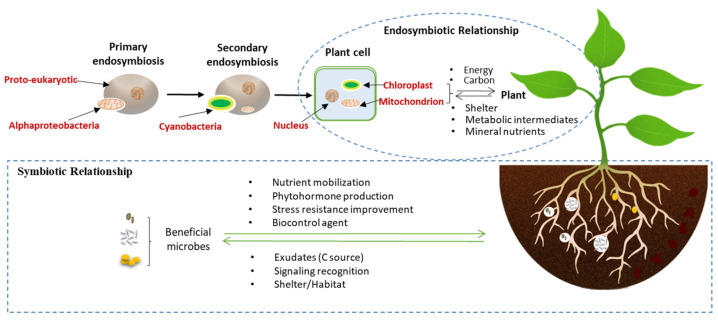
The origin of mitochondria and chloroplasts, as modified prokaryotes that have become plant organelles [8], the endosymbiotic relationship between chloroplast, mitochondrion, and plant itself, and the symbiotic relationships between the beneficial microbes and the host plant. Beneficial microbes include plant growth promoting bacteria and fungi. The shelter and habitat created by the plant host provide a regulated environment and energy source, enhancing the survival of the symbionts and endosymbionts.

The phytomicrobiome is composed of a community of microorganisms that associate and interact with a host plant including bacteria, archaea, fungi, oomycetes, viruses, protozoa, algae, and nematodes [1,2,3]. Collectively, the plant and its phytomicrobiome are a holobiont, a term originally coined by Adolf Meyer-Abich but most frequently associated with and popularized by Lynn Margulis [1] and rigorously explored by Bordenstein and Theis [2]. While the phytomicrobiome includes parasitic and commensal microbes, it is also comprised of mutualists, or beneficial microbes, such as mycorrhizal fungi (MF) and plant growth-promoting bacteria (PGPB) that enable the plant holobiont to survive within a wide range of environments. Beneficial microbes mediate plant holobiont responses to abiotic and biotic stresses and allow the plant holobiont to adapt to environmental variations (Figure 1) [3,4]. The plant host can then modify the abundance and composition of beneficial microbial species within the phytomicrobiome, at least in part, by secreting biochemical compounds. This selection occurs most strongly in the endosphere, followed by the rhizoplane, and finally the rhizosphere [5]. For example, root exudates can select for and promote the growth of certain beneficial microbes by serving as carbon and/or energy sources for microbial metabolism [6]. 

The earliest and arguably most essential example of a specific symbiotic function within the plant holobiome arose from the endosymbiosis of an alpha-proteobacterium and a cyanobacterium. These microbes are now the mitochondrion and chloroplast, respectively, and are microbes that have been fully integrated into plant cells (Figure 1). These endosymbionts did not replace particular functions of the ancestral organism, but rather provided new functions, giving an evolutionarily competitive edge to the newly evolving plants [7].

In this paper, we focus on how beneficial bacteria and fungi, a relatively small fraction of the phytomicrobiome, have had a disproportionately large influence on plant holobiont evolution. We also review the fundamental roles that the phytomicrobiome plays in plant holobiont development and survival. Finally, we propose that a greater integration of holobiont theory should be incorporated into the plant sciences.

## 2. The Plant Holobiont and Its Functional Phytomicrobiome

### 2.1. The Endosymbiosis of Prokaryotes and the Rise of Plant Holobionts

Life on earth is believed to stem from a single origin, the microbial ancestor that emerged as early as 3.5 billion years ago [9,10]. According to endosymbiosis theory, about 1.5 billion years ago, a proto-eukaryotic cell engulfed an alphaproteobacteria, forming an endosymbiotic relationship, and gradually developed into what is now recognized as the mitochondrion (Figure 1) [11]. Mitochondria use alternative electron acceptors to generate ATP and are now the most important organelle for plant respiration since they enable metabolic reactions to convert energy into usable forms. 

Approximately half of a billion years later [12], eukaryotic cells containing mitochondria engulfed cyanobacteria (photosynthetic prokaryotes), which like the alphaproteobacteria became fully incorporated into and dependent on plant cells, resulting in the chloroplast (Figure 1) [13,14,15]. Chloroplasts convert energy from the sun into carbohydrates, using water as the electron donor. However, large-scale gene loss from plastids has occurred during the course of evolution [16], and higher plant chloroplasts now contain only 120–130 genes [12] compared with the 1700 to 7500 genes contained in cyanobacterial genomes [17]. In spite of their reduced genome size, chloroplasts and cyanobacteria still carry out some of the same functions, ranging from gene expression to metabolism [18,19]. For example, it is clear that the protein-targeting system of cyanobacteria is similar to that of the chloroplast [20].

Organisms have been described as entities evolved from constituent elements that are highly cooperative and minimally conflicting; however, there is ongoing debate regarding the levels of cooperation and conflict within holobionts [7,20]. In plants, chloroplasts and mitochondria are highly cooperative with plant cells while relationships between the plant and the phytomicrobiome are more varied including the mutualistic and parasitic interactions. For example, relationships between plants and PGPB are organismal given that they are highly cooperative and low conflict in nature. On the other hand, some plant-microbe interactions are more opportunistic for one member and therefore are not organismal. The plant is therefore a eukaryotic organism, with prokaryotic constituents (entrained microbes), that interacts with its phytomicrobiome to form the plant holobiont [7]. As a result, the difference between the plant and the phytomicrobiome blurs and the concept of the holobiont becomes pre-eminent. It can then be argued that the influence of microbes on the evolution of plants is so profound that without microbes, the concept of the “plant” fails. 

### 2.2. The Phytomicrobiome and the Transition of Plant Holobionts to Terrestrial Environments

The phytomicrobiome helps the plant holobiont survive in a variety of environments (discussed in Section 3). In fact, early in their evolution, plants could not have successfully transitioned from the aquatic environments inhabited by their ancestors without functional support from the phytomicrobiome [21]. The phytomicrobiome has likely been shaped to impart additional genes to the holobiont, therefore altering the niches available to the plant; this allows the plant to adjust its behavior to suit the conditions of its immediate environment. A selective advantage provides the plant holobiont with functional plasticity, allowing it to better access resources and improve its nutrition, growth, and stress tolerance [4]. For further analysis on the roles in which the phytomicrobiome plays in plant holobiont evolution, see several recent reviews [6,22,23,24,25]. 

#### 2.2.1. Beneficial Microbes Help Plant Holobionts Acquire Nutrients 

Following the advent of oxygenic photosynthesis, there was rapid speciation as organisms competed for newly available resources [26]. However, plants have always relied heavily on beneficial microbes to assist in fulfilling their nutrient requirements. Beneficial microbes can support plant holobiont nutrition through 1) biological nitrogen fixation (BNF), 2) solubilization of insoluble nutrients, and 3) increased root surface area.

The role of N_2_ fixation symbioses is primarily to balance the N-cycle for terrestrial plants with their high levels of carbon fixation. There are two categories of N-fixing diazotrophs that produce nitrogenase, the enzyme required for BNF [27,28]: root nodule-forming rhizobia, and free-living bacteria, including cyanobacteria. The root nodule-forming symbiosis involves a relatively small group of plant species, predominantly legumes [29], that form symbiotic associations with rhizobia including *Rhizobium* and *Bradyrhizobium* [30]. There are also associative relationships between free-living nitrogen fixing bacteria and plants that can contribute to plant nitrogen nutrition [31,32,33]. Plant-cyanobacteria symbioses, on the other hand, are found in a wide range of plants including bryophytes, the angiosperm *Gunnera*, and cycads [34].

Almost all plants also form interactions with MF to improve nutrient acquisition. MF can increase the effective root surface area and improve nitrogen, phosphorus, iron, and zinc extraction efficiency from the rhizosphere [35,36]. MF also produce organic acid (e.g., acetic acid, oxalic acid, and succinic acid) exudates that decrease the rhizosphere pH, dissolving insoluble minerals into the soil solution, and contributing to greater nutrient acquisition [35,37,38]. Beyond the already recognized benefits of host associations with MF are substantially broader effects. Simard [37] illustrated that MF facilitate plant “cognition”, allowing the plant host to perceive signals from its surroundings and implement actions that enhance its robustness and overall fitness. In addition, MF promote communication among plants, including with kin and with other organisms, via signaling pathways and therefore contribute to specific changes or overall shifts in plant morphology, physiology, and fitness. Indeed, the evolutionary success of a plant cannot be separated from that of the associated microbes. 

Synergies between members of the plant holobiont microbiome affect nutrient cycling in the rhizosphere and consequently shape plant nutrient status and crop yield in agricultural settings. For example, arbuscular mycorrhizal fungi create a suitable environment for the colonization of plant growth-promoting bacterial endophytes if inoculated together onto crop plants, and plant root and hyphal exudates provide a carbon source for the bacterial endophytes [36,38,39]. Co-inoculation of both fungi and bacteria as a consortium can improve crop yields more than single strain inoculants.

#### 2.2.2. Microbial Phytohormone Production Promotes Plant Holobiont Growth and Stress Resistance

Microbial communities present in the rhizosphere can control plant holobiont growth, development, and stress responses through the production and delivery of plant growth regulators, growth regulatory precursors, or their analogues [40,41]. These plant-microbe interactions rely on a wide variety of long-distance chemical signaling compounds [42] including plant hormones (indole-3-acetic acid (IAA), auxins, cytokinins, and gibberellins) and microbial-produced compounds that can mimic or induce plant hormone production. For example, many bacteria produce auxin or manipulate host auxin signaling to, in the case of rhizosphere PGPB, promote plant root growth, or in the case of plant pathogens, interfere with plant development [43]. A specific example is microbially produced auxins, from either pathogenic or mutualistic bacteria [44], which can influence plant root growth and branching [45]. 

Signal compounds, such as thuricin 17 and lipochitooligosacchardies, produced by beneficial microbes can also assist plant adaptation to biotic and abiotic stresses [46,47]. For example, when plant cells perceive microbial signal compounds, messages are relayed through the plant from stressed tissues to healthy tissues, allowing them to receive “danger” signals, which induce defense-related gene expression [25,48,49]. For more on “danger” signals, see recent reviews [50,51]. Interestingly, beneficial microbes can also induce resistance in the absence of a phytopathogen, which can provide the plant with improved immunity to future phytopathogenic attacks [48]. These interconnected and shared signaling networks play a crucial role in enhancing long-term stress adaptation at the plant holobiont level [50] and allow for complex and integrated defense responses to invaders in a timely manner, which imparts ecological fitness [42]. Similarly, these signal compounds can assist plant defense responses to abiotic stresses including salinity, cold temperature, and drought [47].

#### 2.2.3. Phytomicrobiomes as Biocontrol Agents

Plant holobionts facilitate the development of pathogen-suppressive phytomicrobiomes by selecting for microbial taxa with biocontrol properties. Microbes capable of plant pathogen biocontrol have been widely documented, including numerous cases by strains of *Pseudomonas* spp., *Bacillus* spp., and *Trichoderma* spp. among others [49,52,53,54,55]. In our era of agricultural optimization [56], the possibility of harnessing and augmenting this ancillary plant holobiont immunity highlights the practical implications of the holobiont concept [57,58,59].

Some members of the phytomicrobiome provide either direct or indirect mechanisms of biocontrol. Microbes that release various compounds possessing antimicrobial properties constitute direct mechanisms. Indirect mechanisms of biocontrol limit the fitness of plant pathogens by reducing their ability to access vital resources. Depending on the pathogens, these key resources can include metals and immunologically vulnerable plants. The metal depletion biocontrol mechanism is accomplished through the excretion of siderophores, which chelate soil metals such as iron, copper, and zinc, and funnel them back to the excreting cells using active transport systems [60,61]. The subtraction of the vulnerable host biocontrol mechanism is accomplished via the production of volatile organic compounds (VOCs) that lead to the establishment of induced systemic resistance (ISR) in infected plants [62,63].

There are nevertheless abundant indications of the existence of other holobiont biocontrol systems. All signs point, for example, to phytomicrobiome diversity being key in reducing pathogenic infection efficiency [64,65,66]. This phenomenon inherently suggests the existence of yet-to-be-identified pathogen adversaries, a hypothesis supported by analyses of rhizosphere microbiome responses to pathogen-induced root exudation [67,68], analyses of microbiome networks [69], and genomic/metagenomic analyses [70,71,72]. There is also abundant evidence for the existence of microbial biocontrol mechanisms. Genomic and metagenomic analyses have identified many putatively novel pathogen-antagonistic genes in known biocontrol microbes and suppressive soils [73,74]. Analyses of species-specific and community-wide microbial VOCs have also singled out many compounds that may have the same role [75,76]. Moreover, VOCs produced by microbes can act as plant growth promoters and signaling molecules between plant holobionts and their rhizosphere communities [77]. Furthermore, as signals, VOCs can be transferred via mycorrhizal networks in the rhizosphere between plants and their neighbors [78]. The production and roles of VOCs are complex and indicated a wide range of roles within the rhizosphere; more information on microbial VOCs can be found in several recent reviews [79,80,81]. 

## 3. The Evolution of Plant Holobionts

Natural selection acts upon the phenotype of the holobiont, resulting in changes to the hologenome, or the pooled genomes of the phytomicrobiome and the plant host. Thus, the evolutionary unit of selection is the holobiont [3,82]. For coevolution to occur within a holobiome, both the host and the microbiome must evolve in response to the same environmental pressure(s). This coevolution has resulted in the establishment of complex signaling pathways and feedback loops that use phyto- and microbial-derived compounds to achieve functional regulation of the plant holobiont [83]. For example, root exudates recruit beneficial microbes that release compounds, such as the phytohormone IAA, that promote plant growth and encourage the release of more root exudates, thereby promoting holobiont success [84]. Other exudates, such as flavonoids, act as signaling molecules between plants and their symbionts, for instance to encourage nodule formation, as seen in the co-evolution between the plant family Fabaceae and rhizobia [25,85,86,87,88]. However, evolution of the host and its associated microbiome can still occur independently [89] as in the case of a demibiont, where an organism evolves to associate with another, but the latter is not evolutionarily affected [90].

Holobiomes develop and fluctuate in response to various biogeographical influences. Understanding both microbial and community ecology is necessary when studying plant holobiomes and the interactions that occur within the phytomicrobiome [6,91]. The genetics of the host plant, the genetics of phytomicrobiome, and abiotic and biotic influences all drive the evolution and assembly of the plant holobiont. A greater understanding of how dispersal, selection, drift, and diversification shape microbial communities can clarify how they associate with the plant host [92]. For example, microbial immigration plays a significant role in the diversity of a phytomicrobiome and allows the holobiont to adapt to local environmental changes [93]. However, evolutionary pressures select for plant hosts able to exert control over their phytomicrobiome. If the phytomicrobiome is left unregulated, an increasing randomness of the microbial community composition could be deleterious to host fitness, and evolution would select against the resulting holobiont [94].

The selective pressures that drive holobiont evolution may occur on many levels [90], with some affecting the phytomicrobiome but not their host plant or vice versa, making it unclear whether the holobiont should be considered a biological individual or an ecosystem. The conclusion depends on the perspective taken, either that of the microbe or the macrobe; indeed, it may be both simultaneously [95]. Current arguments support holobionts as biological individuals [96], and indeed this may be the best conclusion to date for both understanding and conserving the natural world [97]. This allows for the exciting possibility of empirically studying hologenomic adaptations which may differ from species-level genomic adaptations [98]. Limiting the definition of the hologenome to only that of the host plant genome and specific traits encoded by core genes of the phytomicrobiome may make possible the study of hologenomic adaptation [98]. This is supported by the idea that natural selection acts on genomic islands, or symbiosis islands, coding for specific symbiotic functions rather than for specific microbial species [93,99].

Microbial communities that are consistently associated with individuals of a eukaryotic species are known as the core microbiome [100]. Elucidating a core phytomicrobiome depends as much on its definition (functional vs. taxonomic) as on the ecological scale of interest (from the individual to the population level) [100]. A functional core microbiome, comprised of taxa required for maintaining host homeostasis, may be different from a strictly taxonomically conserved core microbiome [4]. However, to date it is challenging to interpret functional meaning from metagenomic data alone [101,102,103]. To further complicate matters, the predicted diversity of a core phytomicrobiome is expected to decrease as ecological scope is expanded. As a result, a more general microbial community consisting of fewer taxa that are conserved across a large ecological scale, for example across a plant population, may have a greater role in maintaining survival of the host [4]. In contrast, the remainder of the phytomicrobiome, termed the accessory microbiome, is influenced by the local environment and is not conserved across populations of a given plant species. This explains at least some of the variability in phytomicrobiomes across environments [4].

An eco-holobiont, which encompasses the interactions of a holobiome with the environment and other holobiomes (e.g., holobionts containing other plant or animal species), may blur the already tenuous line regarding what constitutes a holobiont. However, the concept of the eco-holobiont may also help explain discrepancies in the core phytomicrobiomes of a given host species at different ecological scales since it portrays the phytomicrobiome as more fluid than previously considered [5]. For example, the linkages between soil, plant, and pollinator microbiomes exemplify how multiple microbiomes must be considered in unison to gain a complete understanding of community assemblage and dynamics [104]. Further research into plants as holobionts will aid in elucidating the range of microbial relationships that contribute to the ecology and evolution of a species.

## 4. Unculturable Microbes: The Hidden Fraction of the Plant Holobiont 

While we now recognize the integral importance of the phytomicrobiome in plant holobiont function, we have only scratched the surface in our understanding of both their diversity and roles in plant holobiont development. Part of this understanding stems from the realization that a vast majority of microbes in the natural world cannot be cultured using traditional microbiological methods. Over years of extensive studies, it has been consistently found that just 1–5% of microbes are culturable, although this ratio is controversial [105,106,107]. As phytomicrobiome members typically interact with multiple other microbial species, the lack of culturable bacteria complicates our understanding of their exact roles in plant holobiont success [89]. 

The cause for the high proportion of unculturable microbes stems from the complexity and the dynamic conditions of their natural habitat, which are difficult to recreate under laboratory conditions. These unculturable species likely depend on and play important roles within their communities [108,109]. In fact, it is likely that these relationships drove the development of the plant holobiont itself over time, shaping it and the ecological roles found within. While advances in microbiological techniques have allowed researchers to accurately estimate microbial diversity, these techniques alone are unable to elucidate the intricacies of how all members of the phytomicrobiome interact within the plant holobiont [108,110].

## 5. Conclusions

Under natural conditions, plants are always associated with a substantial, diverse, and to some extent regulated population of microbes; together the host plant and the phytomicrobiome constitute the plant holobiont. As we have discussed, the beneficial microbes of the phytomicrobiome provide a range of functional benefits to the plant component of the holobiont, including improved access to nutrients, regulation of plant hormone levels to stimulate growth, and pathogen control. Because the plant cannot survive without the functions provided by the phytomicrobiome, we consider the holobiont to be the entity that evolution acts upon. In the context of agriculture, we also recognize that crop productivity and yield is dependent on holobiont, not just plant, success.

Mitochondria and chloroplasts are derived from ancient plant-microbe interactions that led to the endosymbioses and incorporation of these microbes into integral components of the modern plant cell. Therefore, these examples straddle the line between being members of the phytomicrobiome and organelles of the plant. Thus, rather than view them as one or the other, it would be simpler and perhaps more accurate to consider them as part of the holobiont, acknowledging their inherent dual status. Much in the same way as taxonomy is an anthropomorphic desire to make sense of biodiversity, the conceptualization of plants as biological units is merely our misinformed account of reality. The subject of our discussion, according to the evidence presented, would be better understood as a plant holobiont to fully capture the biological complexity found in nature.

The holobiont and hologenomic concepts have been slow to take hold in the field of plant science compared to other disciplines in the life sciences [111]. For example, the fields of coral biology and animal gut microbiology are readily describing their study systems as holobionts [112,113]. Plant holobionts, like coral holobionts, are functionally sessile in nature and thus heavily rely on their associated phytomicrobiomes [4]. In fact, the field of plant–microbe interactions may perhaps be a misnomer in that it dichotomizes the plant and phytomicrobiome into mutable units. This distinction is not meant to undermine molecular studies that seek to tease apart plant–microbe interactions and are key to understanding them as holobionts, but rather is a suggestion to utilize the concept of the holobiont whenever possible.

Nevertheless, there are a few recent studies in which the plant and its phytomicrobiome are viewed as a holobiont [114]. Experiments in plant ecology and evolution have begun to incorporate the phytomicrobiome into their design; however the hosts and microbes are still considered as separate entities [115]. These research areas should be among the first to incorporate the holobiont concept since evolutionary pressures act upon the holobiont [111]. Another field of research that could greatly benefit from this concept is plant agriculture. The booming field of microbial biostimulants, which aims to promote plant growth while simultaneously reducing the application of synthetic fertilizers and pesticides, often faces the recurring problem where successful laboratory and greenhouse trials do not lead to increased crop yields in field trials [116,117]. The gnotobiotic conditions of proof-of-concept experiments cannot accurately capture the complexity of crop holobionts, let alone their role within the greater eco-holobiont. The plant sciences, and especially the area of plant–microbe interactions, will need to make this paradigm shift in order to fully and accurately incorporate -omics data which are becoming more accessible to researchers seeking to decipher the natural world or have a positive impact on humanity.

## Data Availability

Not applicable.
